# Case Report: Myocarditis Associated With COVID-19 mRNA Vaccination Following Myocarditis Associated With *Campylobacter Jejuni*

**DOI:** 10.3389/fcvm.2022.837759

**Published:** 2022-03-18

**Authors:** Nobuko Kojima, Hayato Tada, Hirofumi Okada, Shohei Yoshida, Kenji Sakata, Soichiro Usui, Hiroko Ikeda, Masaki Okajima, Masa-aki Kawashiri, Masayuki Takamura

**Affiliations:** ^1^Department of Cardiovascular Medicine, Kanazawa University Graduate School of Medical Sciences, Kanazawa, Japan; ^2^Diagnostic Pathology, Kanazawa University Hospital, Kanazawa, Japan; ^3^Department of Emergency Medicine, Kanazawa University Hospital, Kanazawa, Japan

**Keywords:** cardiomyopathy, left ventricle, chest pain, myocarditis, COVID-19

## Abstract

We herein present our experience with a case involving a 17-year-old Japanese boy suffering from acute myocarditis after his second coronavirus disease-2019 (COVID-19) messenger RNA (mRNA) vaccine shot. The patients had a history of myocarditis associated with *Campylobacter jejuni* 3 years prior. This has been the first-ever documented case of myocarditis associated with COVID-19 mRNA vaccination in a patient with a history of myocarditis. We present a series of images and blood biomarkers for different types of myocarditis that developed in this single patient.

## Learning objective

History of myocarditis may be a risk factor for COVID-19 mRNA vaccine-associated myocarditis. Thus, vigilance is required for patients with such a history when considering indications for COVID-19 mRNA vaccination, especially among young boys.

## Introduction

Myocarditis, the main cause of which is viral infections such as coronavirus disease 2019 (COVID-19), is a rare condition, wherein signs of inflammation can be observed in the myocardium (1, 2). Studies have shown that other conditions such as nonviral infections, autoimmune syndromes, and vaccines can also cause myocarditis (1). Soon after the introduction of COVID-19 mRNA vaccination, many case reports exhibiting acute myocarditis associated with the vaccination had emerged (3–5). Accumulated data appear to suggest that the occurrence of myocarditis is more frequent among young adult and adolescent males (6–8). However, it remains unclear whether other risk factors, particularly a history of myocarditis, are present for this condition. Given the current global situation caused by the COVID-19 pandemic, additional data regarding this issue, especially among younger individuals, need to be accumulated. We herein present the first-ever documented case of acute myocarditis associated with COVID-19 mRNA vaccination in a patient who had a history of myocarditis.

## Case Description

### History of Presentation

A 17-year-old Japanese boy, with chest pain occurring 2 days after his second COVID-19 mRNA vaccination (BNT 162b2, manufactured by Pfizer and BioNTech), was presented to a previous hospital. Electrocardiography showed ST elevation in V2 to V5 leads (Supplemental Material). Moreover, his serum cardiac enzymes, including cardiac troponin T (1.605 ng/ml, normal range ≤0.014 ng/ml) and creatinine kinase (CK, 462 IU/L, normal range 62–287 IU/L) were elevated. He was then referred to Kanazawa University Hospital for further investigation and treatment of his chest symptom.

### Medical History

**Figure 1:**
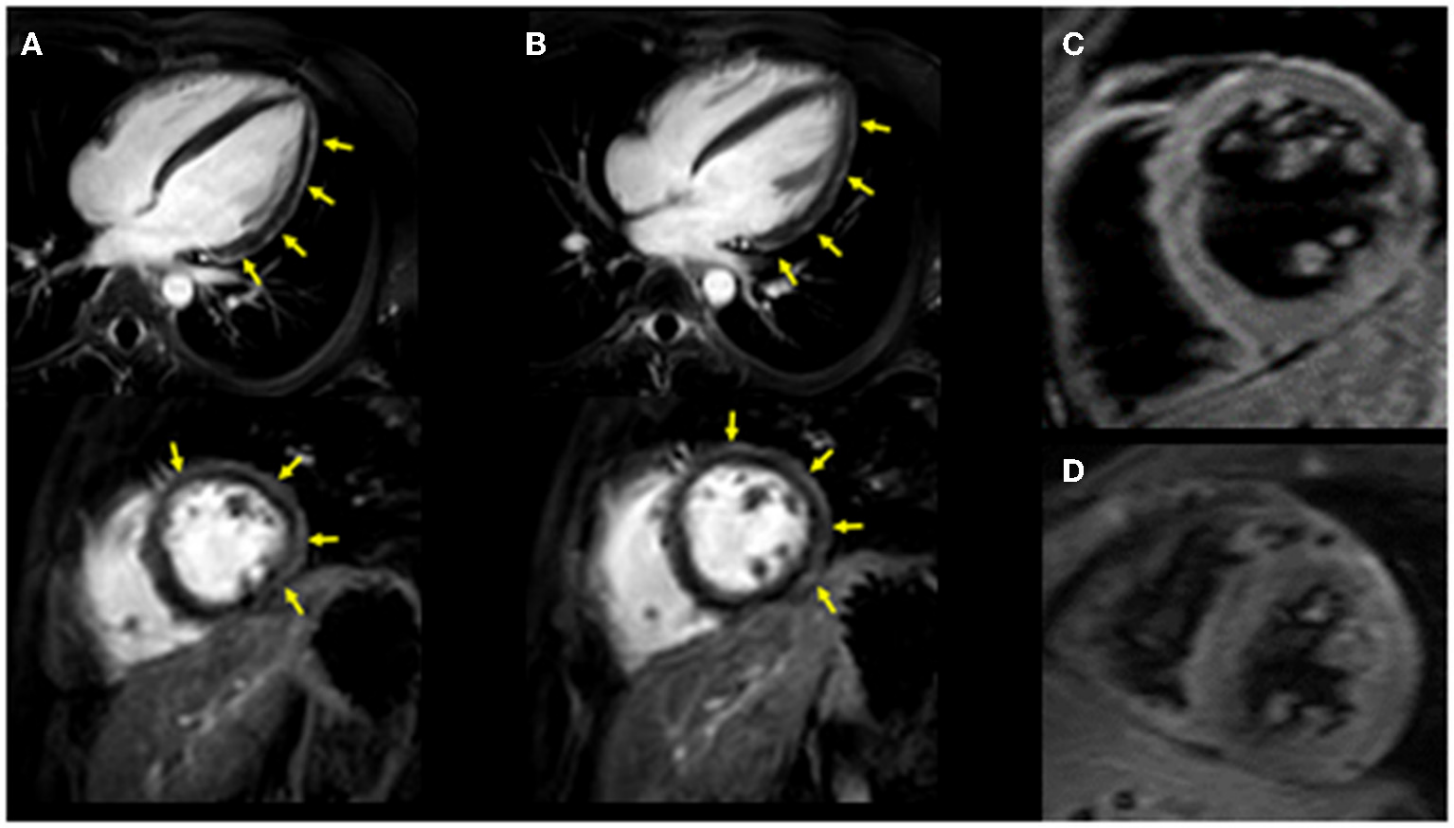
Cardiac MRI imaging. Diffuse late gadolinium enhancement at the epicardium was observed in both images. **(A)** Images obtained 3 years ago when he suffered from his previous myocarditis (top: long-axis view, bottom: short-axis view). **(B)** Images obtained during the current myocarditis episode associated with coronavirus disease-2019 (COVID-19) messenger RNA (mRNA) vaccination (top: long-axis view, bottom: short-axis view). T2-weighted MR images. **(C)** Image obtained 3 years ago when he suffered from his previous myocarditis episode. **(D)** Image obtained during the current myocarditis episode associated with COVID-19 mRNA vaccination.

The patient had a history of myocarditis (causative bacteria was *Campylobacter*) when he was 13 years old, which was treated with intravenous immunoglobulin (IVIG). His initial symptoms included fever, chest pain, and diarrhea. His maximum CK was 1,682 IU/L. Cardiac MRI revealed diffuse late gadolinium enhancement at the epicardium (Figure 1A). A cardiac biopsy was not performed. After the introduction of IVIG, his symptoms improved, for which he was discharged from the hospital without any apparent cardiac dysfunction assessed by echocardiography and myocardial scintigraphy. Enalapril 5 mg/day was introduced and was discontinued 1 year after this episode. He received regular follow-up at our institute, during which, his serum cardiac enzymes were assessed, and electrocardiography and echocardiography were performed. No signs of recurrence had been observed until his last visit 6 days before his second COVID-19 mRNA vaccination. Echocardiography revealed a normal left ventricular ejection fraction (LVEF = 75%), without other dilatations in any chambers, and his cardiac troponin T level was within the normal range (0.006 ng/ml) in his last visit (6 days before his second COVID-19 mRNA vaccination).

### Differential Diagnosis

Acute coronary syndrome and acute systolic heart failure of any cause were considered as differential diagnoses.

### Diagnostic Assessment

**Figure 2:**
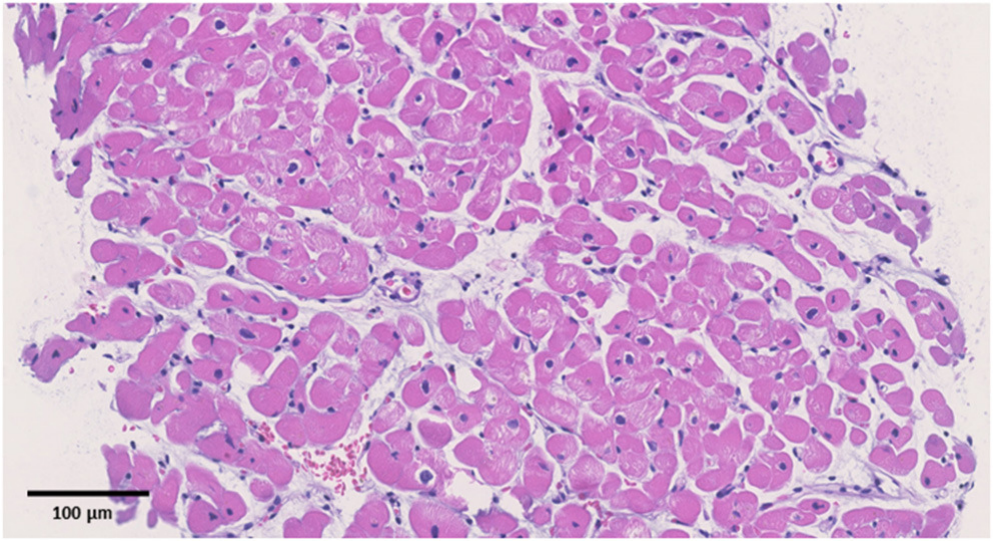
Pathological specimens. Hematoxylin and eosin staining (original magnification ×200). The black bar indicates 100 μm. No apparent signs of inflammation were observed.

Upon admission to our hospital, the patient had blood pressure, heart rate, and body temperature of 135/65 mmHg, 97 bpm, and 37.3°C, respectively. Chest radiography showed no signs of cardiomegaly or pulmonary congestion. Blood tests revealed an elevation in white blood cells (9,560/μl) and C-reactive protein (4.44 mg/dl, normal range ≤0.3 mg/dl), together with elevations in cardiac enzymes, including CK (818 IU/L, normal range 62–287 IU/L), CK-MB (59 IU/L, normal range 2–21 IU/L), and cardiac troponin T (1.41 ng/ml, normal range ≤0.014 ng/ml). The N-terminal pro-brain natriuretic peptide (NT-pro BNP) level was also elevated (221.2 pg/ml, normal range ≤ 125 pg/ml). Electrocardiography revealed ST elevations in V2–V5 leads, whereas echocardiography revealed systolic dysfunction (LVEF = 55%) associated with left ventricular dilatation (LVDd, 55 mm) without any pericardial effusion. Coronary CT showed no signs of coronary atherosclerosis. A myocardial specimen obtained from the septum of the right ventricle showed no apparent signs of myocardial destruction or inflammation (Figure 2). Hemodynamic evaluation by Swan–Ganz catheterization revealed a pulmonary artery pressure of 27/11 (19) mmHg, pulmonary capillary wedge pressure of 13 mmHg, and cardiac output of 6.62 L/min. Cardiac MRI revealed diffuse late gadolinium enhancement at the epicardium (Figure 1B) that was similar but somewhat different from the images observed 3 years prior when he suffered from his previous myocarditis (Figure 1A). A T2-weighted MRI revealed diffuse high-intensity areas, suggesting edematous changes in the left ventricle during his previous bout of myocarditis, as well as during the current myocarditis (Figures 1C,D). Enzyme-linked immunosorbent assays of sera were all negative for potential causes of viral myocarditis (Coxsackie, echo, influenza A and B, cytomegalovirus, and Epstein-Barr virus (EBV)]. Negative T waves were observed in V3 to V6 leads following electrocardiography on day 5 (Supplemental Material). All the aforementioned results, except for pathological findings from the myocardial specimen, were consistent with a diagnosis of COVID-19 mRNA vaccination-related myocarditis. We ruled out acute coronary syndrome given the absence of cardiac asynergy and cardiac MRI findings. We also ruled out acute systolic heart failure of any cause based on the hemodynamic evaluation findings by Swan–Ganz catheterization.

### Management

**Figure 3:**
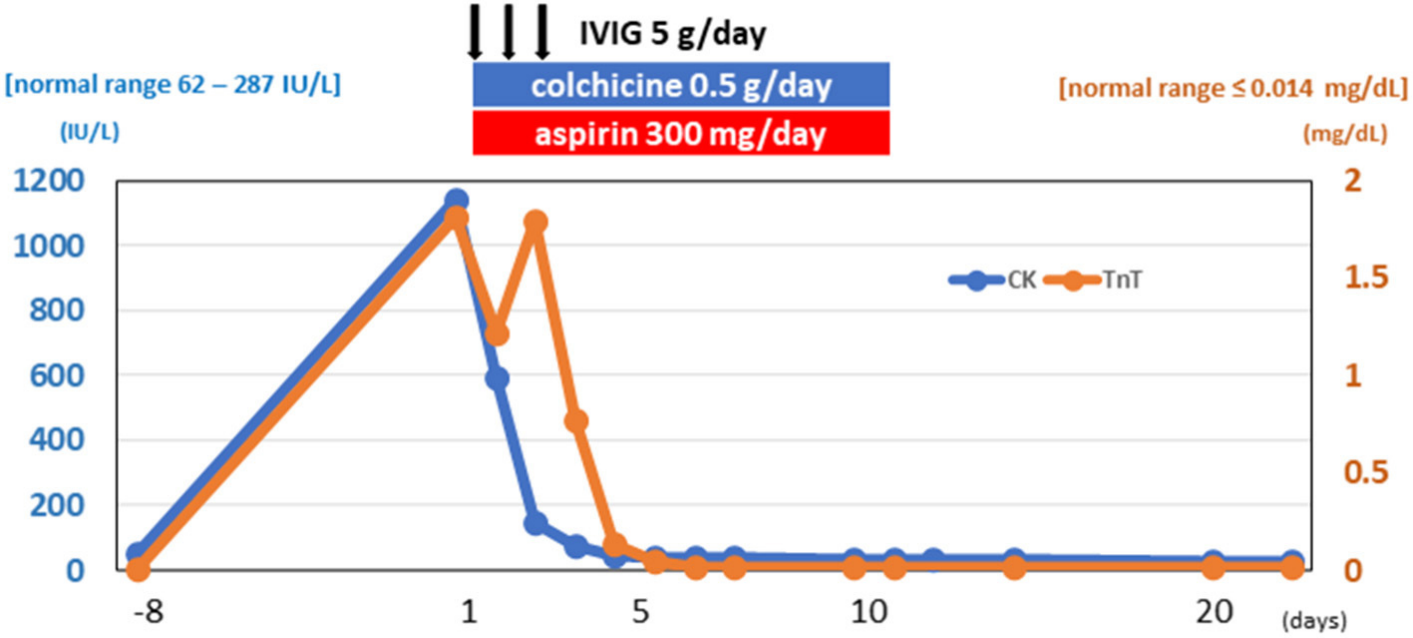
Clinical course. Elevated cardiac enzymes decreased during his clinical course. CK, creatinine kinase; TnT, cardiac troponin T; IVIG, intravenous immunoglobulin.

The patient was started on IVIG treatment (5 g/day × 3 days), colchicine (0.5 g/day × 14 days), and aspirin (300 mg/day × 14 days) (Figure 3). The Japanese guideline (9) utilized by our institute has no clear first-choice therapy for this situation. Among several potential medical therapies, we opted to use IVIG to avoid complications when using immunosuppressive agents. The CK, CK–MB, cardiac troponin T, and NT-proBNP levels gradually returned to normal, and follow-up echocardiography showed normal cardiac function (LVEF = 68%). After being hospitalized for a total of 23 days, the patient was discharged without any symptoms.

## Discussion

Currently, myocarditis is being recognized as one of the complications of COVID-19 mRNA vaccination (1–3). Albeit rare, the prognosis of this condition seems to be quite good. Nonetheless, more information on risk factors for this unfavorable phenomenon needs to be collected (6–8). So far, epidemiological studies have suggested that this condition is more frequently observed among young adult and adolescent males (6–8). However, it is unclear whether a history of other types of myocarditis can be considered a risk factor. In this report, we present the first-ever documented case of myocarditis associated with COVID-19 mRNA vaccination in a patient who had a history of myocarditis (Supplemental Material). Based on a series of investigations, including cardiac enzymes, electrocardiogram, echocardiography, and cardiac MRI, we found similarities between COVID-19 mRNA vaccination-related myocarditis and myocarditis associated with *Campylobacter jejuni*. We observed unique yet similar patterns on cardiac MRI wherein diffuse late gadolinium enhancement was located mainly at the epicardium during both the current COVID-19 mRNA vaccination-related myocarditis and the previous myocarditis episode associated with *Campylobacter jejuni*. Cardiac MRI has been considered a useful modality for diagnosing acute myocarditis (10, 11) given its great potential for not only diagnosis but also understanding of the pathophysiological mechanism of COVID-19 mRNA vaccination-related myocarditis (12–14). There are several limitations to be considered. First, we obtained three specimens at the time of endomyocardial biopsy. Although the patient had no apparent signs of myocardial destruction or inflammation from the endomyocardial biopsy, a diagnosis of myocarditis was established because of his elevated cardiac troponin T, elevated creatinine kinase, reduced EF, changes in the electrocardiogram, and MRI findings. Second, we could not determine the causal association between the history of myocarditis and the current vaccination-associated myocarditis. Third, we did not compare the cardiac MR images between the previous and current myocarditis episodes. Thus, the diffuse late gadolinium enhancement at the epicardium observed during the current myocarditis episode may not have represented acute myocarditis. However, we observed edematous changes in the myocardium using T2-weighted MR images. In addition, the area of late gadolinium enhancement at the epicardium observed in the current myocarditis episode was somewhat different from that of the previous one. These facts support the notion that late gadolinium enhancement at the epicardium observed in the current episode represents acute myocarditis. Lastly, we were unable to perform the suggested immunohistochemical testing on our biopsy specimens to investigate whether there were any autoantibodies against the myocardium. The second episode might, indeed, be associated with post-infectious autoimmune syndrome; however, this situation has been described as a chronic condition rather than an acute one with complications in multiple organs (1). Of note is that the mechanism of myocarditis induced by mRNA vaccination remains unclear. In most cases without a history of previous myocarditis, molecular mimicry between the spike protein of virus and self-antigens, trigger of pre-existing dysregulated immune pathways in certain individuals, immune response to mRNA, activation of immunologic pathways, and dysregulated cytokine expression have been proposed (8). However, in this case with a history of myocarditis, there may be something more in addition to these common mechanisms, although observations from a single case cannot produce any concrete evidence.

In conclusion, special attention may be needed when introducing COVID-19 mRNA vaccination to individuals who have a history of myocarditis. Cardiac MRI can be useful for diagnosing COVID-19 mRNA vaccination-related myocarditis.

## Patient Perspective

We suggest that this episode would not have any serious impact on his cardiac function. However, we will advise the patient to avoid the booster COVID-19 mRNA vaccine because of this episode.

## Data Availability Statement

The datasets presented in this article are not readily available because patients data are not available except for the requests based on legal measures. Requests to access the datasets should be directed to HT, ht240z@sa3.so-net.ne.jp.

## Ethics Statement

Ethical review and approval was not required for the study on human participants in accordance with the local legislation and institutional requirements. Written informed consent to participate in this study was provided by the participants' legal guardian/next of kin.

## Author Contributions

NK, HT, HO, SY, KS, SU, HI, MO, M-aK, and MT contributed to the patient's care and contributed to the preparation of the manuscript. All authors approved the final version of the manuscript.

## Conflict of Interest

The authors declare that the research was conducted in the absence of any commercial or financial relationships that could be construed as a potential conflict of interest.

## Publisher's Note

All claims expressed in this article are solely those of the authors and do not necessarily represent those of their affiliated organizations, or those of the publisher, the editors and the reviewers. Any product that may be evaluated in this article, or claim that may be made by its manufacturer, is not guaranteed or endorsed by the publisher.

## Abbreviations

CK: creatinine kinase
IVIG: intravenous immunoglobulin
MRI: magnetic resonance imaging
LVEF: left ventricular ejection fraction.
