# Supplementary material for: Case Report: Myocarditis Associated With COVID-19 mRNA Vaccination Following Myocarditis Associated With Campylobacter Jejuni
Source: Front Cardiovasc Med. 2022 Mar 18;9:837759. doi: 10.3389/fcvm.2022.837759 (PMC8971558; doi:10.3389/fcvm.2022.837759)

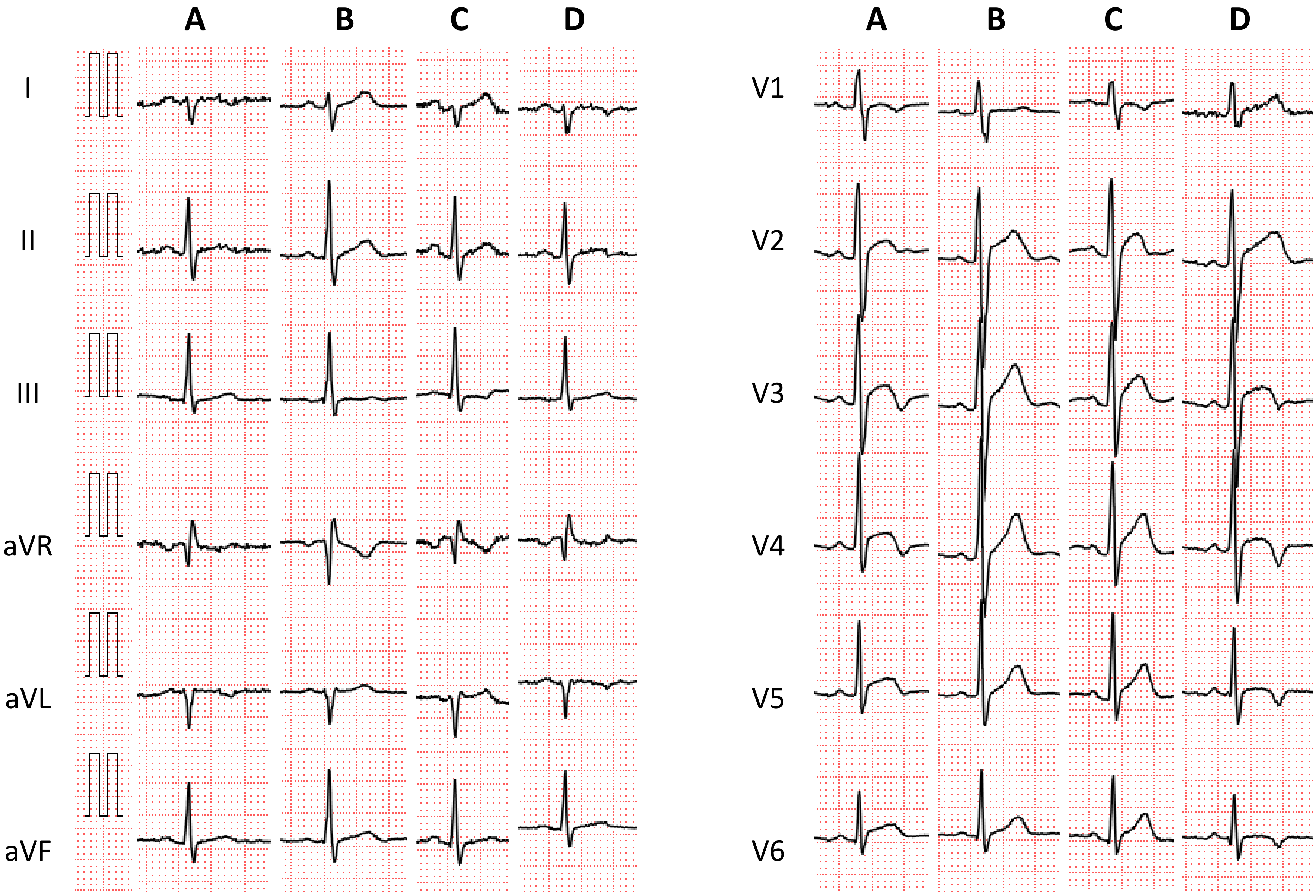

A. At the onset of the previous myocarditis (13-years old)
B. 6 days before the second COVID-19 mRNA vaccination (day –8)
C. 2 days after the second COVID-19 mRNA vaccination (day 0)
D. Day 5

Clinical course of this case
(myocarditis associated with *Campylobacter jejuni* and with Covid-19 mRNA vaccination)

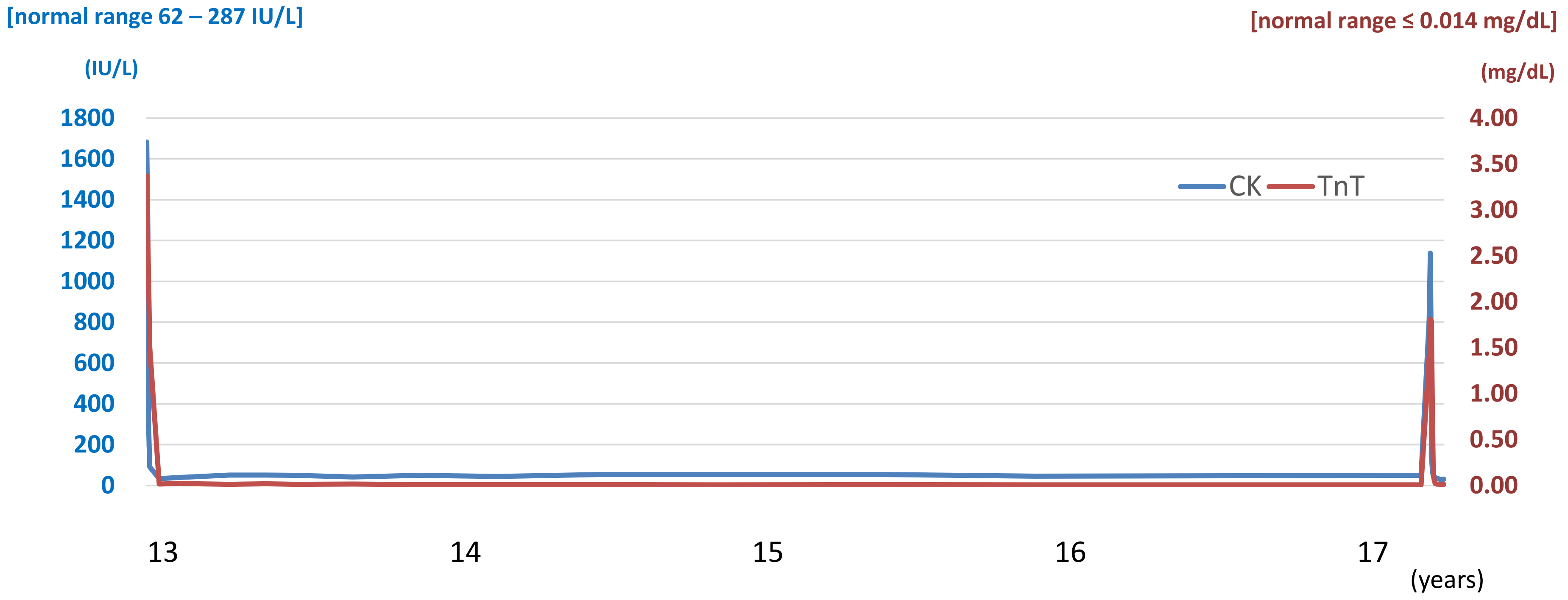

Supplement: Supplementary file 1 [file Data_Sheet_1.pdf]
